# Prevalence, Species Distribution, and Antimicrobial Susceptibility Patterns of Clinically Significant Methicillin-Resistant Coagulase-Negative Staphylococci Isolates From Patients in a Tertiary Care Hospital in Eastern India: A Retrospective Study

**DOI:** 10.7759/cureus.106532

**Published:** 2026-04-06

**Authors:** Amresh Pati, Swaranika Mandal, Kumudini Panigrahi, Khusish Patel, Basanti Kumari Pathi

**Affiliations:** 1 Microbiology, Kalinga Institute of Medical Sciences, Bhubaneswar, IND

**Keywords:** antimicrobial susceptibility, coagulase-negative staphylococci, methicillin resistance, nosocomial infections, tertiary care hospitals, vancomycin

## Abstract

Background

Coagulase-negative *Staphylococci* (CoNS), also referred to as *Staphylococci* other than *Staphylococcus aureus*(SOSA), have emerged as important nosocomial pathogens and are frequently resistant to methicillin, leading to the emergence of methicillin-resistant CoNS (MRCoNS). The present study aimed to evaluate the prevalence, species distribution, and antimicrobial susceptibility patterns of clinically significant MRCoNS isolates from patients in a tertiary care hospital in Eastern India.

Methods

This retrospective, laboratory-based observational study was conducted in the Department of Microbiology at a tertiary care hospital in Eastern India, after getting IEC approval (KIIT/KIMS/IEC/2441/2026). Laboratory and clinical data were collected from records from January 2021 to December 2025 for MRCoNS-positive cultures. Data on species distribution, antimicrobial susceptibility patterns of MRCoNS isolates, and corresponding clinical findings were retrieved from medical records and analyzed. Clinically significant CoNS isolates from patients aged >18 years, all sexes, from various clinical specimens were included in the study. Identification and antimicrobial susceptibility testing were performed using the VITEK 2 compact automated system (bioMérieux, Marcy‑l'Étoile, France). Methicillin resistance was determined using both Oxacillin/cefoxitin disc diffusion and the VITEK 2 system (bioMérieux, Marcy‑l'Étoile, France).

Demographic, clinical, and laboratory data were collected and analyzed using R software Version 4.4.3 (R Foundation for Statistical Computing, Vienna, Austria). Descriptive statistics were used to determine the prevalence. Categorical data were presented as frequencies and percentages. The differences between the prevalence of MRCoNS and MSCoNS were evaluated using the Chi-square test. A p-value of <0.05 was considered statistically significant.

Results

Out of 10,984 clinical isolates of CoNS, 6,326 (57.6%) were confirmed to be MRCoNS. During the five-year period (2021-2025), a steady rise in MRCoNS prevalence was found from 17% to 23%. *S. hemolyticus* (37.9%, n = 2,397), *S. hominis* (21.0%, n = 1,330), and *S. epidermidis* (19.7%, n = 1,244) were the most common MRCoNS species. MRCoNS isolation was higher in ICU patients (60.5%) with male predominance. Specimen-wise, the highest proportions of MRCoNS were seen in blood (37%) and urine (35%), with 5% and 22% in sterile body fluids and others, respectively. Reduced susceptibility was found to oxacillin, erythromycin, ciprofloxacin, levofloxacin, clindamycin, and cotrimoxazole, whereas all MRCoNS isolates remained susceptible to vancomycin, teicoplanin, and linezolid.

Conclusion

This study shows a steady upsurge in the prevalence of MRCoNS during the stipulated time period, with *S. hemolyticus* as the predominant type. Even though MRCoNS remained susceptible to last-resort drugs like vancomycin, linezolid, and teicoplanin, stringent antimicrobial stewardship practices and improved infection control measures are recommended to reduce the spread of MRCoNS in hospitals as well as to prevent further development of resistance.

## Introduction

Staphylococcal infections are still one of the most common types of healthcare-associated infections (HAIs) around the world. They cause a lot of illness, death, and higher healthcare costs [1]. *S. aureus*, especially its methicillin-resistant variant (MRSA), has historically been the principal subject of clinical and epidemiological research; however, coagulase-negative *Staphylococci* (CoNS) have become increasingly significant nosocomial pathogens [2].

CoNS, previously regarded as contaminants or harmless commensals of the skin and mucous membranes, are now acknowledged as genuine pathogens capable of inducing severe infections, especially in immunocompromised individuals and patients with implanted foreign bodies [3]. This paradigm shift shows how well they can make biofilms on medical devices like central venous catheters, prosthetic joints, and heart valves, which makes infections hard to treat and likely to come back [4].

The most clinically significant CoNS species are *S. epidermidis* (the most common), *S. hemolyticus*, *S. hominis*, and *S. saprophyticus* [5]. *S. epidermidis* is often linked to device-related infections, while *S. hemolyticus* is particularly distinguished by its significant prevalence of multidrug resistance [6].

Methicillin resistance in CoNS, facilitated by the mecA gene that encodes penicillin-binding protein 2a (PBP2a), represents a significant clinical issue as CoNS act as a genetic reservoir for resistance genes capable of horizontal transfer to more virulent pathogens such as *S. aureus* [7,8]. This phenomenon underscores the importance of keeping an eye on MRCoNS in healthcare settings from an epidemiological point of view.

India is experiencing a significant upsurge of antimicrobial resistance (AMR), with one of the highest global burdens of methicillin-resistant *Staphylococci* [9]. Recent findings from an Indian study highlight this trend: in a cohort of individuals with bloodstream infections at a tertiary-care hospital in Raipur, CoNS, predominantly *S. hemolyticus*, were identified as true pathogens in 2.35% of sepsis cases. Most of the isolates did not respond to methicillin, although they did respond to vancomycin, teicoplanin, and linezolid [10]. Prior studies in India have indicated varying prevalence rates of MRCoNS, ranging from 32.7% to 62.7%, characterized by notable geographic and institutional disparities [11,12]. A recent international study of non-coagulase *Staphylococcus* isolates from Iraq revealed that all clinical isolates developed biofilm and exhibited elevated rates of multidrug resistance, frequently harboring mecA [13]. This diversity underscores the imperative for localized epidemiological data to guide institution-specific infection control and antibiotic stewardship policies.

MRCoNS are resistant to beta-lactams and often resistant to fluoroquinolones and macrolides. However, they have always been susceptible to glycopeptides (vancomycin and teicoplanin) and oxazolidinones (linezolid) [14,15]. Nevertheless, recent reports indicating diminished vancomycin susceptibility and sporadic linezolid resistance in global contexts evoke apprehensions regarding the future therapeutic landscape [15]. Despite the growing clinical significance of MRCoNS, there is a paucity of long-term, species-specific data from Eastern India.

Therefore, the present study was undertaken to determine the prevalence, species distribution, antimicrobial susceptibility patterns, and temporal trends of clinically significant methicillin-resistant CoNS (MRCoNS) isolates from patients in a tertiary care hospital in Eastern India.

## Materials and methods

Study design

This was a retrospective, laboratory-based observational study carried out in the Department of Microbiology of a tertiary care hospital in Bhubaneswar, Eastern India. After obtaining approval from the Institutional Ethics Committee (KIIT/KIMS/IEC/2441/2026), laboratory culture and antimicrobial susceptibility data, as well as corresponding clinical data, were retrieved from medical records for positive MRCoNS isolates from patients who were admitted to our hospital between January 2021 and December 2025.

All specimens had been collected, processed, and tested previously in the microbiology laboratory as part of routine diagnostic workup. Only the corresponding laboratory and clinical records were retrieved and analyzed.

Study population

Clinically significant isolates of CoNS obtained from infected adult patients (age >18 years) of either sex and isolates from clinical specimens (blood, urine, wound exudates, body fluids, and other relevant samples) were included. Recurrent isolates from the same patient within a 14‑day period, isolates categorized as contaminants, samples from non‑sterile sites without accompanying clinical indicators of infection, and records with missing clinical data were excluded from the study.

Study procedure

Clinical specimens such as blood, urine, body fluids, and other samples (e.g., catheter tips, exudates, and swabs) were collected using aseptic techniques and sent promptly to the microbiology laboratory. They were processed immediately using standard microbiological procedures. Direct smears were prepared from each specimen for Gram staining. Blood samples were inoculated into BACT‑ALERT 3D (bioMérieux, Marcy‑l'Étoile, France) blood culture bottles and incubated at 37°C for up to five days in the automated system. Sterile body fluid specimens were inoculated onto blood agar, MacConkey agar, and chocolate agar plates, and 1-3 mL of each specimen was also inoculated into pediatric blood culture bottles of the BACT‑ALERT 3D system (bioMérieux, Marcy‑l'Étoile, France). Pus and exudate specimens were cultured on blood agar and MacConkey agar, while urine samples were inoculated onto CLED agar and incubated at 37°C for 24-48 hours.

From positive culture plates and blood culture bottles, subcultures were made to obtain pure isolates. Initial identification was done by gram staining, catalase, and coagulase tests [16]. Further species‑level identification of CoNS was done using the VITEK 2 system (bioMérieux, Marcy‑l'Étoile, France) with GP identification cards, following the manufacturer’s instructions.

Organisms identified as CoNS were considered clinically significant based on standard criteria for each specimen type [4]. For blood, significance required pure growth in both bottles with a differential time to positivity <24 hours, together with at least three clinical criteria (fever >100°F, leukocytosis >12,000/mm³, clinical features of sepsis, systolic blood pressure <90 mmHg, hospital stay >48 hours, or risk factors such as long‑term intravascular catheterization, immunosuppression, central lines, dialysis, or extensive post‑surgical infection). For CSF and other sterile body fluids, any growth of CoNS was considered significant when correlated with Gram stain findings and compatible clinical features. Isolates from wound or tissue samples were considered significant when the Gram stain results correlated with clinical signs of infection. For urine, significant bacteriuria was defined as pure growth of ≥10⁵ CFU/mL from clean‑catch midstream or catheterized specimens.

Detection of MR

Cefoxitin disc diffusion was performed using 30 μg cefoxitin discs (Hi‑Media) on Mueller-Hinton agar plates and incubated at 35-37°C for 16-18 hours. A zone diameter ≤21 mm indicates resistance and ≥22 mm indicates susceptibility according to CLSI M100, 36th edition (2026). Oxacillin resistance was defined as MIC ≥2 μg/mL which were determined using the VITEK 2 AST‑P628 card (bioMérieux, Marcy‑l'Étoile, France) [17]. Isolates resistant by either method were classified as MRCoNS.

Antimicrobial susceptibility testing

Antimicrobial susceptibility test (AST) was done with VITEK 2 AST-P628 card (bioMérieux, Marcy‑l'Étoile, France). The antibiotics present in the panel were cefoxitin, benzyl penicilin, oxacillin, gentamicin, ciprofloxacin, levofloxacin, erythromycin, clindamycin, linezolid, daptomycin, teicoplanin, vancomycin, tigecycline, cotrimoxazole. Interpretation of antibiotic susceptibility was done as per CLSI 2026 cut-off values. Results were interpreted as susceptible, intermediate or resistant according to the CLSI M100, 36th edition (2026) guidelines [17].

Data collection and statistical analysis

Data regarding patient demographics (age and sex) and microbiological culture and sensitivity results (organism and AST profile) were retrieved from electronic medical records and entered into Microsoft Excel (Microsoft Corporation, Redmond, Washington). Statistical analysis was performed using R software Version 4.4.3 (R Foundation for Statistical Computing, Vienna, Austria). Categorical variables were presented as frequencies and percentages, and the prevalence of MRCoNS and MSCoNS was compared using the Chi‑square test; p < 0.05 was considered statistically significant.

## Results

A total of 10,984 clinically significant CoNS isolates were isolated from different clinical samples in the microbiology laboratory during the study period. Of these, 6,326 (57.6%) were MRCoNS and 4,658 (42.4%) were methicillin‑susceptible (MSCoNS).

Table 1 shows that the majority of the CoNS isolates in the present study were from male patients (6259/10984, 57.0%), who also accounted for a higher proportion of MRCoNS (3738/ 6326, 59.1%) compared to female patients (2588/6326, 40.9%) (p < 0.001). Year-wise distribution demonstrated an increasing trend in MRCoNS proportion from 2021 (1892/6326, 17%) to 2025 (1453/6326, 23%), while MSCoNS proportions fluctuated over the years. A statistically significant variation (p < 0.001) was observed among the year-wise prevalence. A statistically significant difference was observed between ICU and non-ICU settings, with MRCoNS and prevalence of 60.5% (2779/4596) and 55.5% (3547/6388), respectively (p < 0.001). Among specimen types, blood showed the highest percentage of MRCoNS (2371/6326, 37%), followed by urine (2205/6326, 35%) and other specimens (1409/6326, 22%), while body fluids had the lowest proportion of MRCoNS (341/6326, 5%), p < 0.001.

**Table 1:** Distribution of MRCoNS and MSCoNS among the study population. Descriptive variables were expressed as frequency and percentage. The Chi-square test was used, and Chi-square values were given along with the p-values. A p-value of <0.05 was considered statistically significant. ICU: intensive care unit; MRCoNS: methicillin-resistant coagulase-negative *Staphylococcus*; MSCoNS: methicillin-sensitive coagulase-negative *Staphylococcus.*

Parameter	Total (n = 10984)	MRCoNS (n = 6326)	MSCoNS (n = 4658)	Statistical test used	Test statistics	p-value
Gender						
Male	6259 (57%)	3738 (59%)	2521 (54%)	Chi-square test	8.382	< 0.001
Female	4725 (43%)	2588 (41%)	2137 (46%)			
Year						
2021	1892 (17%)	1076 (17%)	816 (17%)	Chi-square test	11.297	< 0.001
2022	2089 (19%)	1121 (18%)	968 (21%)			
2023	2552 (23%)	1374 (22%)	1178 (25%)			
2024	2119 (19%)	1302 (20%)	817 (17%)			
2025	2332 (21%)	1453 (23%)	879 (19%)			
Location						
ICU	4596 (42%)	2779 (44%)	1817 (39%)	Chi-square test	10.093	< 0.001
Non-ICU	6388 (58%)	3547 (56%)	2841 (61%)			
Specimen						
Blood	4122 (38%)	2371 (37%)	1751 (38%)	Chi-square test	16.738	< 0.001
Urine	3546 (32%)	2205 (35%)	1341 (28%)			
Body fluid	570 (5%)	341 (5%)	229 (5%)			
Others	2746 (25%)	1409 (22%)	1337 (28%)			

**Figure 1:**
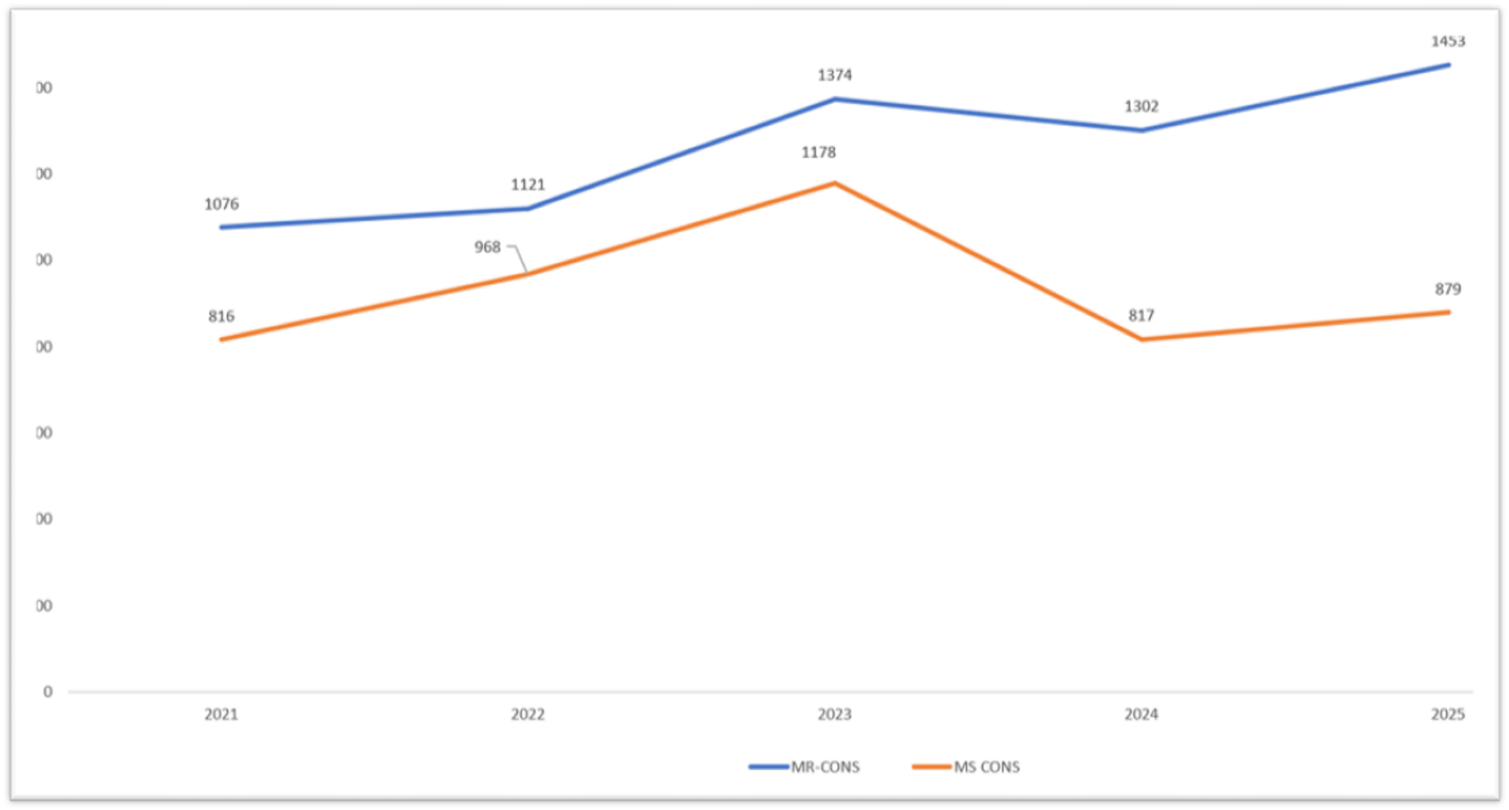
Year-wise distribution of MRCoNS and MSCoNS isolates (2021-2025). MRCoNS: methicillin-resistant coagulase-negative *Staphylococcus*; MSCoNS: methicillin-sensitive coagulase-negative *Staphylococcus.*

Figure 1 shows the annual trend of MRCoNS and MSCoNS isolates from 2021 to 2025. MRCoNS consistently remained higher than MSCoNS throughout the study period. A year-wise analysis of MRCoNS prevalence showed a progressive increase during stipulated time period, rising from 53.7% in 2021 (1076/1892) to 56.9% in 2023 (1374/2552), and further to 61.5% in 2024 (1302/2119) and 62.3% in 2025 (1453/2332) (p < 0.001), indicating a steady escalation in methicillin resistance over time.

Table 2 shows the distribution of MRCoNS species across year, location, and specimen type. Among total MRCoNS isolates, *S. hemolyticus* was predominant species (2397/6326, 37.9%) followed by *S. hominis* (1330/6326, 21.0%) and *S. epidermidis* (1244/6326, 19.7%). Year-wise distribution of the species types showed a gradual increase in all major species, Overall, the proportion of isolates increased from 1076/6326 (17%) in 2021 to a peak of 1453/6326 (23%) in 2025. Most species showed a gradual rising trend, with *S. epidermidis* demonstrating a notable increase in 2025 (328/1244, 26%). Every year, *S. hemolyticus* was the most prevalent type of MRCoNS. Its numbers kept going up, from 428 isolates (18% of all MRCoNS) in 2021 to 520 (22%) in 2025. A statistically significant variation (p< 0.001) was observed among them. With respect to location, most isolates were from non-ICU settings (3547/6326, 56%); however, *S. epidermidis* (47%) and *S. hemolyticus* (46%) were relatively more common in ICU patients compared to other species (p < 0.001). Specimen-wise distribution revealed that blood was the most common source (2371/6326, 37%), with comparable contributions from *S. epidermidis, S. hemolyticus, *and *S. hominis*. Urine samples accounted for 2205/6326 (35%) of isolates, with a predominance of *S. saprophyticus* (189/335, 57%). Body fluids contributed a smaller proportion (341/6326, 5%), while other specimens accounted for 1409/6326 (22%) of isolates. All observed differences were statistically significant (p < 0.001).

**Table 2:** Distribution of subgroups of MRCONS among the study population. Descriptive variables were expressed as frequency and percentage. The Chi-square test was used, and Chi-square values were given along with the p-values. A p-value of <0.05 was considered statistically significant. MRCoNS: methicillin-resistant coagulase-negative *Staphylococcus.*

Parameter	Total (n = 6326)	*Staphylococcus epidermidis* (n = 1244)	*Staphylococcus hemolyticus* (n = 2397)	*Staphylococcus hominis* (n = 1330)	*Staphylococcus saprophyticus* (n = 333)	Other CONS (n = 1022)	Statistical test used	Test statistics	p-value
Year									
2021	1076 (17%)	195 (16%)	428 (18%)	221 (17%)	57 (17%)	175 (17%)	Chi-square test	13.195	< 0.001
2022	1121 (18%)	206 (17%)	447 (19%)	228 (17%)	56 (17%)	184 (18%)			
2023	1374 (22%)	264 (21%)	514 (21%)	293 (22%)	81 (24%)	222 (22%)			
2024	1302 (20%)	251 (20%)	488 (20%)	287 (21%)	66 (20%)	210 (20%)			
2025	1453 (23%)	328 (26%)	520 (22%)	301 (23%)	73 (22%)	231 (23%)			
Location									
ICU	2779 (44%)	586 (47%)	1097 (46%)	575 (43%)	140 (42%)	381 (37%)	Chi-square test	21.379	< 0.001
Non-ICU	3547 (56%)	658 (53%)	1300 (54%)	755 (57%)	193 (58%)	641 (63%)			
Specimen									
Blood	2371 (37%)	472 (38%)	935 (39%)	505 (38%)	81 (24%)	378 (37%)	Chi-square test	51.506	< 0.001
Urine	2205 (35%)	385 (31%)	815 (34%)	479 (36%)	189 (57%)	337 (33%)			
Body fluid	341 (5%)	49 (4%)	144 (6%)	53 (4%)	44 (13%)	51 (5%)			
Others	1409 (22%)	338 (27%)	503 (21%)	293 (22%)	19 (6%)	256 25%)			

Table 3 shows the antimicrobial susceptibility pattern of *S. epidermidis*. Over the course of five years, all isolates (100%) were susceptible to vancomycin, linezolid, and teicoplanin. All isolates remained uniformly susceptible to vancomycin, teicoplanin, and linezolid, with high susceptibility also to daptomycin and tigecycline (≥95%). A declining trend was observed for gentamicin, cotrimoxazole, and fluoroquinolones over the years, with gentamicin susceptibility dropping from 63% in 2021 to 38% in 2025, and cotrimoxazole susceptibility dropping from 65% to 60% over the same time period. Erythromycin and clindamycin showed consistently high resistance (≤20% susceptible).

**Table 3:** AST findings of Staphylococcus epidermidis (n = 1244). AST: antimicrobial susceptibility test; GM: gentamicin; CIP: ciprofloxacin; SXT: cotrimoxazole; VA: vancomycin; LZD: linezolid; DAP: daptomycin; RA: rifampicin; CM: clindamycin; E: erythromycin; TGC: tigecycline; LVX: levofloxacin; TEC: teicoplanin.

Year	GM	CIP	SXT	VA	LZD	DAP	RA	CM	E	TGC	LVX	TEC
Year 2021 (n = 195)												
Susceptible	123 (63%)	103 (53%)	127 (65%)	195 (100%)	195 (100%)	193 (99%)	117 (60%)	35 (18%)	4 (2%)	193 (99%)	103 (53%)	195 (100%)
Intermediate	2 (1%)	33 (17%)	2 (1%)	0	0	2 (1%)	76 (39%)	43 (22%)	64 (33%)	2 (1%)	33 (17%)	0
Resistant	70 (36%)	59 (30%)	66 (34%)	0	0	0	2 (1%)	117 (60%)	127 (65%)	0	59 (30%)	0
Year 2022 (n = 206)												
Susceptible	115 (56%)	105 (51%)	132 (64%)	206 (100%)	206 (100%)	202 (98%)	115 (56%)	41 (20%)	4 (2%)	202 (98%)	101 (49%)	206 (100%)
Intermediate	17 (8%)	35 (17%)	11 (5%)	0	0	4 (2%)	80 (39%)	33 (16%)	66 (32%)	4 (2%)	29 (14%)	0
Resistant	74 (36%)	66 (32%)	63 (31%)	0	0	0	11 (5%)	132 (64%)	136 (66%)	0	76 (37%)	0
Year 2023 (n = 264)												
Susceptible	132 (50%)	129 (49%)	161 (61%)	264 (100%)	264 (100%)	259 (98%)	129 (49%)	45 (17%)	5 (2%)	256 (96%)	124 (47%)	264 (100%)
Intermediate	29 (11%)	45 (17%)	21 (8%)	0	0	5 (2%)	103 (39%)	42 (16%)	82 (31%)	8 (4%)	34 (13%)	0
Resistant	103 (39%)	90 (34%)	82 (31%)	0	0	0	32 (12%)	177 (67%)	177 (67%)	0	106 (40%)	0
Year 2024 (n = 251)												
Susceptible	113 (45%)	118 (47%)	156 (62%)	251 (100%)	251 (100%)	246 (98%)	113 (45%)	43 (17%)	10 (4%)	246 (98%)	113 (47%)	251 (100%)
Intermediate	35 (14%)	38 (15%)	25 (10%)	0	0	5 (2%)	95 (38%)	43 (17%)	70 (28%)	5 (2%)	38 (15%)	0
Resistant	103 (41%)	95 (38%)	70 (28%)	0	0	0	43 (17%)	165 (66%)	171 (68%)	0	95 (38%)	0
Year 2025 (n = 328)												
Susceptible	125 (38%)	151 (46%)	197 (60%)	328 (100%)	328 (100%)	318 (97%)	181 (55%)	46 (14%)	13 (4%)	312 (95%)	131 (40%)	328 (100%)
Intermediate	52 (16%)	46 (14%)	36 (11%)	0	0	10 (3%)	95 (29%)	52 (16%)	85 (26%)	16 (5%)	46 (14%)	0
Resistant	151 (46%)	131 (40%)	95 (29%)	0	0	0	52 (16%)	230 (70%)	230 (70%)	0	151 (46%)	0

Table 4 shows the antimicrobial susceptibility pattern of *S. hemolyticus*. All isolates remained uniformly susceptible to vancomycin, teicoplanin, and linezolid (100% throughout), with high susceptibility also to daptomycin and tigecycline (≥97%). Over the study period, gentamicin and cotrimoxazole susceptibility in *S. hemolyticus* declined (from 67% to 51% and 68% to 64%, respectively), and susceptibility to fluoroquinolones such as ciprofloxacin and levofloxacin remained persistently low (approximately 50-57%). For erythromycin and clindamycin, with resistance reaching up to ~60-70%, was noted.

**Table 4:** AST findings of Staphylococcus hemolyticus (n = 2397). AST: antimicrobial susceptibility test; GM: gentamicin; CIP: ciprofloxacin; SXT: cotrimoxazole; VA: vancomycin; LZD: linezolid; DAP: daptomycin; RA: rifampicin; CM: clindamycin; E: erythromycin; TGC: tigecycline; LVX: levofloxacin; TEC: teicoplanin.

Year	GM	CIP	SXT	VA	LZD	DAP	RA	CM	E	TGC	LVX	TEC
Year 2021 (n = 428)												
Susceptible	287 (67%)	223 (52%)	291 (68%)	428 (100%)	428 (100%)	424 (99%)	300 (70%)	35 (18%)	13 (3%)	424 (99%)	244 (57%)	428 (100%)
Intermediate	13 (3%)	56 (13%)	4 (1%)	0	0	4 (1%)	124 (29%)	43 (22%)	124 (29%)	4 (1%)	56 (13%)	0
Resistant	128 (30%)	149 (35%)	133 (31%)	0	0	0	4 (1%)	117 (60%)	291 (68%)	0	128 (30%)	0
Year 2022 (n = 447)												
Susceptible	268 (60%)	264 (59%)	287 (64%)	447 (100%)	447 (100%)	438 (98%)	277 (62%)	94 (21%)	9 (2%)	433 (97%)	237 (53%)	447 (100%)
Intermediate	27 (6%)	49 (11%)	17 (4%)	0	0	9 (2%)	152 (34%)	49 (11%)	134 (30%)	14 (3%)	58 (13%)	0
Resistant	152 (34%)	134 (30%)	143 (32%)	0	0	0	18 (4%)	304 (68%)	304 (68%)	0	152 (34%)	0
Year 2023 (n = 514)												
Susceptible	298 (58%)	293 (57%)	334 (65%)	514 (100%)	514 (100%)	509 (99%)	288 (56%)	103 (20%)	16 (3%)	504 (98%)	262 (51%)	514 (100%)
Intermediate	36 (7%)	51 (10%)	10 (2%)	0	0	5 (1%)	170 (33%)	46 (9%)	170 (33%)	10 (2%)	51 (10%)	0
Resistant	180 (35%)	170 (33%)	170 (33%)	0	0	0	56 (11%)	365 (71%)	328 (64%)	0	201 (39%)	0
Year 2024 (n = 488)												
Susceptible	269 (55%)	259 (53%)	327 (67%)	488 (100%)	488 (100%)	438 (98%)	259 (53%)	103 (21%)	15 (3%)	438 (98%)	259 (53%)	488 (100%)
Intermediate	25 (5%)	44 (9%)	54 (11%)	0	0	10 (2%)	205 (42%)	58 (12%)	205 (42%)	10 (2%)	54 (11%)	0
Resistant	194 (40%)	185 (38%)	107 (22%)	0	0	0	24 (5%)	327 (67%)	268 (55%)	0	175 (36%)	0
Year 2025 (n = 520)												
Susceptible	265 (51%)	276 (53%)	333 (64%)	520 (100%)	520 (100%)	504 (97%)	265 (51%)	104 (20%)	21 (4%)	505 (97%)	264 (51%)	520 (100%)
Intermediate	42 (8%)	57 (11%)	42 (8%)	0	0	16 (3%)	213 (41%)	94 (18%)	165 (32%)	15 (3%)	57 (11%)	0
Resistant	213 (41%)	187 (36%)	145 (28%)	0	0	0	42 (8%)	322 (62%)	333 (64%)	0	199 (38%)	0

Table 5 shows the antimicrobial susceptibility pattern of *S. hominis*. All isolates remained uniformly susceptible to vancomycin, teicoplanin, and linezolid (100% across all years), with high susceptibility also to daptomycin and tigecycline (≥97%). A declining trend in susceptibility was observed for gentamicin (54% in 2021 to 41% in 2025), ciprofloxacin (51% to 37%), levofloxacin (51% to 41%), and cotrimoxazole (57% to 42%). High resistance was noted for erythromycin and clindamycin, with susceptibility remaining low (≤31%) and resistance increasing up to 66-70% by 2025. Rifampicin showed moderate activity, but with a substantial intermediate fraction.

**Table 5:** AST findings of Staphylococcus hominis (n = 1330). AST: antimicrobial susceptibility test; GM: gentamicin; CIP: ciprofloxacin; SXT: cotrimoxazole; VA: vancomycin; LZD: linezolid; DAP: daptomycin; RA: rifampicin; CM: clindamycin; E: erythromycin; TGC: tigecycline; LVX: levofloxacin; TEC: teicoplanin.

Year	GM	CIP	SXT	VA	LZD	DAP	RA	CM	E	TGC	LVX	TEC
Year 2021 (n = 221)												
Susceptible	119 (54%)	113 (51%)	126 (57%)	221 (100%)	221 (100%)	221 (100%)	119 (54%)	49 (22%)	7 (3%)	119 (99%)	113 (51%)	221 (100%)
Intermediate	13 (6%)	18 (8%)	4 (2%)	0	0	0	91 (41%)	49 (22%)	89 (40%)	2 (1%)	47 (21%)	0
Resistant	89 (40%)	90 (41%)	91 (41%)	0	0	0	11 (5%)	123 (56%)	125 (57%)	0	61 (28%)	0
Year 2022 (n = 228)												
Susceptible	116 (51%)	112 (49%)	116 (51%)	228 (100%)	228 (100%)	228 (100%)	112 (49%)	71 (31%)	18 (8%)	228 (100%)	112 (49%)	228 (100%)
Intermediate	18 (8%)	28 (12%)	9 (4%)	0	0	0	103 (45%)	41 (18%)	91 (40%)	0	43 (19%)	0
Resistant	94 (41%)	88 (39%)	103 (45%)	0	0	0	13 (6%)	116 (51%)	119 (52%)	0	73 (32%)	0
Year 2023 (n = 293)												
Susceptible	138 (47%)	129 (44%)	138 (47%)	293 (100%)	293 (100%)	290 (99%)	138 (47%)	70 (24%)	9 (3%)	290 (99%)	134 (46%)	293 (100%)
Intermediate	12 (4%)	32 (11%)	21 (7%)	0	0	3 (1%)	132 (45%)	38 (13%)	111 (38%)	3 (1%)	36 (12%)	0
Resistant	143 (49%)	132 (45%)	134 (46%)	0	0	0	23 (8%)	185 (63%)	173 (59%)	0	123 (42%)	0
Year 2024 (n = 287)												
Susceptible	126 (44%)	118 (41%)	132 (46%)	287 (100%)	287 (100%)	284 (99%)	132 (46%)	49 (17%)	15 (5%)	281 (98%)	118 (41%)	287 (100%)
Intermediate	17 (6%)	29 (10%)	17 (6%)	0	0	3 (1%)	123 (43%)	49 (17%)	91 (32%)	6 (2%)	43 (15%)	0
Resistant	144 (50%)	140 (49%)	138 (48%)	0	0	0	32 (11%)	189 (66%)	181 (63%)	0	126 (44%)	0
Year 2025 (n = 301)												
Susceptible	123 (41%)	112 (37%)	127 (42%)	301 (100%)	301 (100%)	295 (98%)	133 (44%)	46 (15%)	12 (4%)	292 (97%)	123 (41%)	301 (100%)
Intermediate	21 (7%)	30 (10%)	21 (7%)	0	0	6 (2%)	117 (39%)	45 (15%)	90 (30%)	9 (3%)	30 (10%)	0
Resistant	157 (52%)	159 (53%)	153 (51%)	0	0	0	51 (17%)	210 (70%)	199 (66%)	0	148 (49%)	0

Table 6 shows AST patterns of *S. saprophyticus.* Vancomycin, linezolid, teicoplanin, and tigecycline remained consistently active against all isolates, with 100% susceptibility throughout the five-year period, and daptomycin susceptibility continuously ≥95%. Gentamicin and cotrimoxazole were moderately susceptible but became less so over time (40% and 60%, respectively). The isolates were resistant to ciprofloxacin, levofloxacin, erythromycin, and clindamycin.

**Table 6:** AST findings of Staphylococcus saprophyticus (n = 333). AST: antimicrobial susceptibility test; GM: gentamicin; CIP: ciprofloxacin; SXT: cotrimoxazole; VA: vancomycin; LZD: linezolid; DAP: daptomycin; RA: rifampicin; CM: clindamycin; E: erythromycin; TGC: tigecycline; LVX: levofloxacin; TEC: teicoplanin.

Year	GM	CIP	SXT	VA	LZD	DAP	RA	CM	E	TGC	LVX	TEC
Year 2021 (n = 57)												
Susceptible	32 (56%)	28 (49%)	35 (61%)	57 (100%)	57 (100%)	56 (98%)	37 (65%)	12 (21%)	3 (5%)	54 (95%)	28 (49%)	57 (100%)
Intermediate	3 (5%)	11 (19%)	7 (12%)	0	0	1 (2%)	17 (30%)	12 (21%)	17 (30%)	3 (5%)	7 (12%)	0
Resistant	22 (39%)	18 (32%)	15 (26%)	0	0	0	3 (5%)	33 (58%)	37 (65%)	0	22 (39%)	0
Year 2022 (n = 56)												
Susceptible	28 (50%)	24 (43%)	29 (52%)	56 (100%)	56 (100%)	56 (100%)	28 (50%)	13 (23%)	4 (7%)	52 (93%)	27 (48%)	57 (100%)
Intermediate	4 (7%)	10 (18%)	8 (14%)	0	0	0	15 (27%)	17 (30%)	19 (34%)	4 (7%)	5 (9%)	0
Resistant	24 (43%)	22 (39%)	19 (34%)	0	0	0	13 (23%)	26 (46%)	33 (59%)	0	24 (43%)	0
Year 2023 (n = 81)												
Susceptible	38 (47%)	31 (38%)	40 (49%)	81 (100%)	81 (100%)	77 (95%)	38 (47%)	12 (15%)	4 (5%)	77 (95%)	25 (31%)	81 (100%)
Intermediate	12 (15%)	14 (17%)	9 (11%)	0	0	4 (5%)	31 (38%)	13 (16%)	38 (47%)	4 (5%)	9 (11%)	0
Resistant	31 (38%)	36 (45%)	32 (40%)	0	0	0	12 (15%)	56 (69%)	39 (48%)	0	47 (58%)	0
Year 2024 (n = 66)												
Susceptible	29 (44%)	21 (32%)	31 (47%)	66 (100%)	66 (100%)	64 (97%)	31 (47%)	9 (14%)	6 (9%)	64 (97%)	21 (32%)	66 (100%)
Intermediate	12 (18%)	12 (18%)	8 (12%)	0	0	2 (3%)	25 (38%)	12 (18%)	21 (32%)	2 (3%)	8 (12%)	0
Resistant	25 (38%)	33 (50%)	27 (41%)	0	0	0	10 (15%)	45 (68%)	39 (59%)	0	37 (56%)	0
Year 2025 (n = 73)												
Susceptible	30 (41%)	19 (26%)	30 (41%)	73 (100%)	73 (100%)	70 (96%)	27 (37%)	11 (15%)	6 (8%)	70 (96%)	19 (26%)	73 (100%)
Intermediate	6 (8%)	11 (15%)	8 (11%)	0	0	3 (4%)	19 (26%)	11 (15%)	21 (29%)	3 (4%)	8 (11%)	0
Resistant	37 (51%)	43 (59%)	35 (48%)	0	0	0	27 (37%)	51 (70%)	46 (63%)	0	46 (63%)	0

## Discussion

This retrospective study reveals a high prevalence of MRCoNS (57.6%) in a tertiary care hospital in Eastern India. This finding is consistent with earlier reports from the region mentioning the prevalence rate of 32.7% and 62.7% [14,18,19]. The high prevalence found in our study indicates that MRCoNS is a significant clinical and epidemiological concern in our setting. In this study, MRCoNS were commonly isolated from male patients (59%). This male predominance may be due to increased exposure to risk factors such as invasive procedures or conditions like prostatic disease requiring urinary catheterization. Similar findings have been reported by Patil G et al. [10]. The steady rise in MRCoNS prevalence from 17% to 23% (2021-2025) suggests increasing selection pressure and persistence of resistant strains. These findings underscore the need for strengthened antimicrobial stewardship and infection control practices [20].

In our study, a significant proportion (44%) of MRCoNS was isolated from ICU patients (p < 0.001), likely due to prolonged hospitalization, invasive device use, and immunocompromised status, biofilm-associated infections leading to methicillin resistance [21,22]. However, the considerable proportion (56%) from non- ICU areas indicates that MRCoNS is not limited to critical care settings and may reflect widespread antimicrobial pressure and device use across the hospitals. Findings of our study are similar to those of studies shown by Singhai M et al. [22] and Saint S et al. [23]. The present study documented *S. hemolyticus* as the most common MRCoNS species (37.9%), followed by *S. hominis* (21.0%) and *S. epidermidis* (19.7%). The distribution of MRCoNS isolates in our study findings is in line with what other countries and Indian studies have found [6,10,13].

*S. epidermidis* is the most common CoNS species that has been isolated for a long time, since it is common on human skin and may easily contaminate blood cultures. In our study, it is found to be the third most common pathogen, largely connected to infections caused by medical devices [24]. The percentage of *S. epidermidis* that has grown over time (from 16% to 26%) shows that things are changing, presumably because of nosocomial transmission. However, the overall ecology stays the same, with no one species becoming dominant.

*S. hemolyticus* was less common in most studies, but it was very common in our analysis, with urinary (34%) and other (mainly wound specimens) (22%) categories. This is consistent with its known ability to cause urinary tract infections and wound infections. This species is especially worrisome because it is often linked to multidrug resistance and lower susceptibility to glycopeptides in certain investigations, even though our isolates were completely susceptible [25]. The relative distribution of species we saw in our study is a little different from what other worldwide studies say. This could mean that the types of specimens, the demographics of the patients, or the geography or climate have an effect on staphylococcal epidemiology [26].

Blood and urine samples exhibited the highest prevalence of MRCoNS (37% and 35%, respectively), likely reflecting clinical severity, device-associated infections (particularly *S. epidermidis*), and possible inclusion of contaminants despite the strict criteria [13,27]. A high proportion of isolates in urine supports the emerging role of CoNS in nosocomial urinary tract infections [28]. In contrast, lower prevalence in sterile body fluids (5%) may be due to fewer invasive interventions or differences in organism virulence [29]. AST results of our study show that all MRCoNS isolates were 100% susceptible to vancomycin, linezolid, and teicoplanin. This total susceptibility to these last-resort agents offers essential therapeutic alternatives for clinicians treating severe MRCoNS infections, contrasting sharply with the rising resistance trends noted in certain international centers [30,31].

The MRCoNS isolates in our study showing complete susceptibility to vancomycin are particularly noteworthy in light of the well-documented occurrence of "vancomycin MIC creep," which refers to the gradual elevation of minimum inhibitory concentrations to vancomycin among *Staphylococci*, even in the absence of clinical treatment failures [32]. Similar high susceptibility has been documented in Indian studies, suggesting preserved efficacy of glycopeptide antibiotics [10,14,18]. However, high resistance to commonly used agents such as erythromycin and fluoroquinolones is concerning, as it limits the oral and outpatient treatment options [14]. The high prevalence of multidrug resistance highlights the growing AMR burden, often necessitating the use of reserved drugs like vancomycin, linezolid, and teicoplanin [33].

Strengths and limitations

Strengths of this study include its large sample size, five‑year observation window, and detailed species‑ and specimen‑stratified AST data, which complement and extend existing baseline national and international MRCoNS datasets. Further, these findings provide important baseline data on MRCoNS epidemiology in an Eastern India tertiary care hospital, which opens the door to future prospective, multicenter studies. Our study has several limitations. Its retrospective design limited control for confounders and precise attribution of clinical outcomes to MRCoNS versus concurrent infections or comorbidities. Device exposure could not be systematically documented; some contaminant misclassification is possible (particularly *S. epidermidis* from non‑sterile sites). Findings may not generalize beyond our single Eastern Indian tertiary center, and molecular characterization of resistance determinants was not performed.

## Conclusions

This study demonstrates a high and increasing prevalence of MRCoNS (57.6%) in a tertiary care hospital, with a rising trend from 2021 to 2025. MRCoNS were frequently associated with infections among ICU patients and showed a rising trend from 53.7% to 62.3% over time, although a substantial proportion from non-ICU areas indicates widespread distribution across the hospital. *S. hemolyticus*, *S. hominis*, and *S. epidermidis* were the predominant species. All isolates remained uniformly susceptible to vancomycin, linezolid, and teicoplanin, while high resistance to commonly used antibiotics such as β-lactams, fluoroquinolones, and macrolides reflects a multidrug-resistant pattern. These findings highlight the growing burden of MRCoNS and underscore the need for strengthened antimicrobial stewardship, infection control measures, and continued surveillance to limit the spread of resistance.
